# Nutrient and Energy Apparent Digestibility of Protein-Based Feed Ingredients and Effect of the Dietary Factors on Growth Performance and Feed Utilization of Sobaity Seabream, *Sparidentex hasta*

**DOI:** 10.3390/ani14060933

**Published:** 2024-03-18

**Authors:** Seemab Zehra, Joseph Leopoldo Q. Laranja, Aboobucker Siddik Abulkasim, Reda Saleh, Paulo H. De Mello, Edoardo Pantanella, Jorge Alarcon, Abdulaziz M. Al-Suwailem, A. Al Shaikhi, Brett D. Glencross, Asaad H. W. Mohamed

**Affiliations:** 1 Kaust Beacon Development, King Abdullah University of Science and Technology, Thuwal, Jeddah 23955, Saudi Arabia; joseph.laranja@kaust.edu.sa (J.L.Q.L.); aboobucker.abulkasim@kaust.edu.sa (A.S.A.); reda.azam@kaust.edu.sa (R.S.); paulo.demello@kaust.edu.sa (P.H.D.M.); edoardo.pantanella@neom.com (E.P.); jalarcon@openblue.com (J.A.); abdulaziz.alsuwailem@kaust.edu.sa (A.M.A.-S.); 2 Oceanography Department, Faculty of Science, Alexandria University, Alexandria 5424041, Egypt; 3 Ministry of Environment, Water and Agriculture, King AbdulAziz Rd., Riyadh 11195, Saudi Arabia; ali.alshaikhi@mewa.gov.sa; 4 Institute of Aquaculture, University of Stirling, Stirling FK9 4LA, UK; brett.glencross@gmail.com

**Keywords:** *Sparidentex hasta*, feeds, apparent digestibility coefficient, protein ingredient, growth performance

## Abstract

**Simple Summary:**

The diet development process for new species includes various steps. Included among these is the investigation of the potential of different feed ingredients so that diets can be formulated with some flexibility to utilize a wider range of resources. Another step is the evaluation of existing diets (designed for other species) when fed to the new species being studied. The sobaity seabream, *Sparidentex hasta*, is considered a promising fish species for aquaculture in the Arabian Gulf region because of its good adaptation to culture, rapid growth, and high market value. This study investigated the responses to three existing commercial diets on the performance of the fish. Additionally, the digestibility of various locally available protein feed ingredients was also examined with the species. Our findings provide quantitative nutrient digestibility data for different feed ingredients and benchmark the responses of the fish to some locally available commercial diets. These findings provide a foundation to begin formulating nutritionally balanced diets for sobaity seabream from resources found locally.

**Abstract:**

Two separate feeding trials were undertaken to benchmark a series of commercial diets and determine the nutrient and energy apparent digestibility coefficients of a variety of protein-based feed ingredients when fed to sobaity seabream, *Sparidentex hasta*. In Experiment 1, triplicate groups of fish (initial body weight: 330.5 ± 2.6 g) were fed with one of three locally available diets containing crude protein (CP) levels ranging from 44 to 46% of dry matter (DM), each with ~12% crude fat. Fish grew at around 3.2 g day^−1^ with a specific growth rate (SGR) of 0.7% day^−1^. Both the feed conversion ratio (FCR) and protein efficiency ratio (PER) were significantly better in fish fed diets, which contained the highest (46.4%) crude protein level. Overall, the data from these preliminary studies suggest that the best performance by sobaity seabream was obtained with a diet containing 46% crude protein, 20 MJ/kg, and a protein-to-energy ratio of 23 mg/kJ. In Experiment 2, fish with an initial body weight of 319 ± 7 g were held in 11 tanks and fed reference (D1) and test diets (D2–D11) for 7 days before fecal collection. This process was repeated twice in a blocking arrangement to generate three replicates. Each of the ten test diets contained 30% of a test ingredient, with the remaining 70% proportionally identical to the D1 diet. Diet apparent digestibility coefficients (ADCs) were measured, and the diet ADCs were then used to derive the protein and energy ADCs for the individual test ingredients. Ingredient protein ADC ranged between 75.5 and 93.9%, while ingredient energy ADC ranged between 66.8 and 81.2%.

## 1. Introduction

The sobaity seabream, *Sparidentex hasta*, is considered a promising fish species for aquaculture in the Arabian Gulf region because of its good adaptation to culture, rapid growth, and high market value [1]. The species is considered to have a high potential for aquaculture in the Red Sea; therefore, several studies have been initiated to look at various biological aspects of its farming [2,3,4,5,6,7,8]. Studies that address the effects of dietary needs for the species are part of that development, but at present, the available information is limited. As such, a preliminary assessment of some existing commercial diets was considered as a first step to evaluate some of the primary responses by the species.

Another critical step in the diet development process includes the investigation of potential feed ingredients for the species. This remains a priority due to the global pressures on fish meal and fish oil and the need to maintain flexibility to utilize a wider range of feed ingredients [9]. This is especially so for carnivorous species, which have traditionally relied on marine ingredients to provide much of their dietary protein and energy demands. As formulators push to reduce reliance on fish meal and fish oil by using terrestrial animal- and plant-based ingredients, they are also endeavoring to restrict protein and fat content to levels that satisfy but do not exceed the animals’ nutrient requirements. Apparent digestibility coefficients (ADCs) provide an objective assessment of the nutrient availability of feeds and can be used to screen ingredients so that diets can subsequently be formulated on a digestible nutrient and energy basis. Being able to formulate on a digestible nutrient and energy basis allows for better optimization of the nutritional value and reduction in the cost of formulated diets. The digestibility of a feed ingredient depends primarily on its chemical composition and the digestive characteristics of the species to which it is fed [10]. Digestibility studies underpin the ability to understand the nutritional potential of both diets and, by inference, ingredients. With modern diets being formulated on a digestible nutrient and energy basis, without this assessment included early on in the assessment of any ingredient, the subsequent assessment of ingredients can become flawed and lead to erroneous assessments [11].

In order to pave the way for the production of sobaity seabream under conditions prevailing in the Red Sea area, this study was undertaken to analyze the potential of existing commercial diets available in the Kingdom of Saudi Arabia on the performance of sobaity seabream. Additionally, this study also assessed the digestibility of various locally available protein feed ingredients by this fish species so that nutritionally balanced diets could be subsequently formulated.

## 2. Materials and Methods

### 2.1. Experiment 1: Diet Benchmarking

#### 2.1.1. Fish Management

Fish were obtained from either a commercial producer or the Jeddah Fisheries Research Center (JFRC; Jeddah, Saudi Arabia) and held in holding tanks (2000 L), being fed a commercial diet (MarineFish; ARASCO, Al Kharj, Saudi Arabia), for several weeks before being acclimated to circular experimental tanks (850 and 1100 L). The flow-through tank system supplied filtered marine water (salinity ~42 PSU) with dissolved oxygen typically around 7.4 ± 0.3 mg/L (mean ± S.D.) at a flow rate of about 5 L/min to each of the tanks. To ensure that the fish were disease-free, the health status of the fish was determined by submitting fish samples to the Fish Health and Safety Laboratory of the Jeddah Fisheries Research Center, Jeddah, Kingdom of Saudi Arabia, for pathogen and parasite analyses before starting the trial. Fish were tested using qPCR for viral nervous necrosis (VNN), red sea bream iridovirus (RSIV), *Streptococcus iniae* (SI), and *Streptococcus agalactiae* (SA), and no infections were recorded. The fish were maintained in indoor holding tanks until they reached the size needed for the feeding trials.

In Experiment 1, fish with an average weight of 330.5 ± 2.6 g were randomly distributed into triplicate groups of 34 fish held in nine circular tanks (850 L). Each tank of fish was fed with one of three locally available diets for 80 days, with the diets containing crude protein levels ranging from 44 to 46% dry matter (DM) and each with ~12% crude fat. The proximate and mineral compositions of the commercial diets are presented in Table 1 and Table 2. Each of the tanks was filled with filtered seawater (salinity c. 41–42‰; ambient water temperature, 26–30 °C) under a flow-through system at a flow rate of 5 L min^−1^. Assessment of the fish was conducted every 2 weeks to determine weight gain and survival. Assessment took place in the early morning, and feeding was withheld until the following day. Fish were anesthetized using AQUI-S (20 ppm) prior to handling, with fish being allowed to recover in a holding tank before being returned to their allocated tank after assessment. All the fish in each tank were weighed individually.

#### 2.1.2. Calculation of Growth Parameters 

At the end of the experiment, the following variables were calculated with the following formulas:

Survival (%) = (Final number of fish/Initial number of fish) × 100

Total weight gain (g/fish) = Final weight − Initial weight

Specific growth rate (%/day) = 100 × (ln final weight − ln initial weight)/days of culture

Feed intake (g/fish) = Total feed consumed per tank/Total fish per tank

Protein efficiency ratio (PER) = Weight gain ÷ total protein fed

Feed conversion ratio (FCR) = Dry feed intake/Wet body weight gain

Daily growth index (%/day) = (Final weight)^1/3^ − (Initial weight^1/3^) × 100)/(Days of culture)

Thermal growth coefficient (%/degree − day) = [FBW^1/3^ − IBW^1/3^] × 1000/[mean temperature × days of culture]

#### 2.1.3. Water Quality Parameters

Water quality parameters were measured daily during the feeding trials [12], and the following data were observed: temperature varied from 25.9 to 30.4 °C (Experiment 1) and 24 to 32 °C (Experiment 2), dissolved oxygen 6.1–7.8 mg L^−1^, nitrate 1.8–1.84 mg L^−1^, ammonia 0.012–0.042 mg L^−1^, and pH 7.3–8.1. Each of the water quality parameters was analyzed using a ProDSS Multiparameter Digital Water Quality Meter, YSI—a Xylem brand, Yellow Springs, OH, USA.

#### 2.1.4. Chemical Analyses

From Experiment 1, six subsamples of each of the experimental diets and samples of the initial and final fish were analyzed to determine their chemical composition using AOAC methods [13]. Crude protein was measured by the Kjeldhal × 6.25 method (AOAC, 2005; Method 960.52). Samples were oven-dried to estimate the moisture content using a thermostat ([13] Method 925.10). Crude fat was assessed using an NMR technique after checking the selected calibration. The intensity of the NMR signal obtained from the dried sample (MQC-23-35 pulsed NMR system-Oxford Instruments, Tubney Woods, Abingdon, UK [13]; Method 2003.05). Ash content was estimated using AOAC methods ([13] 923.03). The amino acid profiles ([13] 984.27) and the mineral and trace elements ([13] 984.27, EN 15763) of the diets were also determined. All samples were submitted and analyzed at the Australian Laboratory Services Arabia Company Ltd. (ALS Arabia, Jeddah, Saudi Arabia). The gross energy contents of the diets were calculated using the following caloric values for proteins (23.6 MJ kg^−1^), lipids (39.5 MJ kg^−1^), and carbohydrates (17.3 MJ kg^−1^) [14].

### 2.2. Experiment 2: Digestibility Assessment

#### 2.2.1. Diet Development

A survey was undertaken from July to November 2017 of three feed mills in the Kingdom of Saudi Arabia to review the range of proteinaceous and lipid ingredients available to the feed industry in the country. Samples of key ingredients were obtained from each of these commercial feed mills and retained for analysis and feed production (Table 3). Samples of the ingredients were sent to a toll commercial feed manufacturer (SPAROS Lda, Olhão, Portugal) for further processing (hammer milling) and feed production. Additional ingredients were provided by SPAROS Lda as reference ingredients. A basal mash was formulated based on standard diet specifications for marine fish and prepared to include approximately 46% protein, 12% fat, and an inert marker (yttrium oxide at 0.1%) (Table 4). The basal mash was prepared and thoroughly mixed, forming the basis for all experimental diets in Study 2. The feed ingredients (Fish Meal (UMP, Sparos), Fish Meal (ARASCO, Riyadh, Saudi Arabia), Fishmeal 3 (LT70 Norvik, Sopropêche, Wimille, France), Tuna Meal (Sparos), Corn Gluten Meal (ARASCO), Soybean Meal (Sparos), Soybean Meal (ARASCO), Wheat Meal (Naqua, Jeddah, Saudi Arabia), Wheat Gluten (Naqua), and SPC (Soycomil, ADM Animal Nutrition, Quincy, IL, USA)) of study for each test diet were added at 30% inclusion, with a concomitant subsample of the basal mash used to make up 100% of the diet mix, respectively (Table 4).

#### 2.2.2. Fish Management

In Experiment 2, 25 fish with an average weight of 319 ± 7 g were randomly distributed into each of 11 circular tanks (1100 L). Each tank was fed a different experimental diet (D1–D11) for seven days before fecal collection. The proximate compositions of the experimental diets are presented in Table 4. Each of the tanks was filled with filtered seawater from the Red Sea (salinity c. 41–42‰; ambient water temperature, 27–30.6 °C) under a flow-through system and had a flow rate of 5 L min^−1^. This process was repeated twice to generate three replicates of each treatment over time.

Each tank of fish was hand-fed to apparent satiety once daily every 30 min over a two-hour period (09:00–11:00). After feeding, any uneaten feed was collected daily by sieving outflow water from the tank standpipe and weighted to calculate the amount of feed intake to provide an indicative assessment of the palatability of the respective feed ingredients. The amount of feed intake per day for all the treatments ranged between 2.3 and 3.2 g/fish/day. Following the seven-day acclimation period, feces were collected from each fish within each tank using stripping techniques based on those reported by Glencross and Hawkins [14]. Fish were netted from their respective tank and placed in a smaller aerated tank containing AQUI-S (20 ppm) until they lost consciousness. Fish were monitored until they lost equilibrium, and as they entered a state of muscle relaxation, they were removed from the water. The feces were then removed from the distal intestine using gentle abdominal pressure. Hands were rinsed between handling each fish. Care was maintained to ensure that the feces were not contaminated by pseudofeces, urine, or mucous. After removal of the feces from the fish, the fecal sample was placed in a small plastic vial and stored in a freezer at −20 °C. Stripped feces were collected between 16:00 h and 18:00 h, with each fish only being stripped twice and not on consecutive days. Fecal samples from different days were pooled within the tank and kept frozen before being freeze-dried in preparation for analysis.

#### 2.2.3. Chemical and Digestibility Analyses

For Experiment 2, all analytical work was undertaken by ChemCentre (Bentley, WA, Australia). Diet, ingredient, and fecal samples were analyzed for dry matter, yttrium, nitrogen, starch, ash, lipid, and energy content. Dry matter was calculated by gravimetric analysis following oven drying at 105 °C for 24 h. Total yttrium concentrations were determined after acid digestion using inductively coupled plasma atomic emission spectrophotometry. Protein levels were calculated from the determination of total nitrogen by an elemental analyzer based on *N* × 6.25. Total lipids were determined gravimetrically following extraction of the lipids using the chloroform–methanol solubilization method. Gross ash content was determined gravimetrically following the loss of mass after combustion of a sample in a muffle furnace at 550 °C for 12 h. Gross energy was determined by adiabatic bomb calorimetry. Total carbohydrates were calculated based on the dry matter content of a sample minus the protein, lipid, and ash. Diet (DADC) and ingredient (IADC) apparent digestibility coefficients were measured and calculated respectively according to the following formula:

DADC_Nutr_ = 1 − (Y_diet_ × Nutr_faeces_)/(Y_faeces_ × Nutr_diet_)

where Y_diet_ and Y_faeces_ represent the yttrium content of the diet and feces, respectively, and Nutr_diet_ and Nutr_faeces_ represent the nutritional parameter of concern (dry matter, protein, starch, lipid, or energy) content of the diet and feces, respectively. The digestibility values for each of the test ingredients in the test diets examined in this study were calculated according to the following formula:

$${IADC}_{ingredient}=\frac{\left({AD}_{test}\times {Nutr}_{test}-\left({AD}_{basal}\times {Nutr}_{basal}\times 0.7\right)\right)}{\left(0.3\times {Nutr}_{ingredient}\right)}$$

where Nutr × AD_ingredient_ is the digestibility of a given nutrient from the test ingredient included in the test diet at 30%. AD_test_ is the apparent digestibility of the test diet. AD_basal_ is the apparent digestibility of the basal diet, which makes up 70% of the test diet. Nutr_Ingredient_, Nutr_test_, and Nutr_basal_ are the levels of the nutrient of interest in the ingredient, test diet, and basal diet, respectively [15]. All raw material inclusion levels were also corrected for dry matter contribution and the effects that this may have had on the actual ratio of reference diet to test ingredient.

Ingredient digestibilities greater than 100% were not corrected because we consider them as potentially indicative of interactive effects between the diet and the test ingredient and should be stipulated as determined.

### 2.3. Statistical Analysis

Prior to statistical analysis, all data were tested for normality and equality of variances by means of Shapiro–Wilk and Levene’s tests, respectively. Normally distributed data were analyzed using a one-way ANOVA followed by Fisher’s least significant difference test (LSD test) [16] to compare significant differences between treatments. Data that failed the normality and equal variance tests were analyzed using the Kruskal–Wallis H test and subsequently analyzed using the Student–Newman–Keuls (SNK) test to compare significant differences (*p* < 0.05) between treatments. The software OriginPro 2020 (San Clemente, CA, USA) was used to employ all the statistical analyses.

## 3. Results

### 3.1. Experiment 1

**Figure 1:**
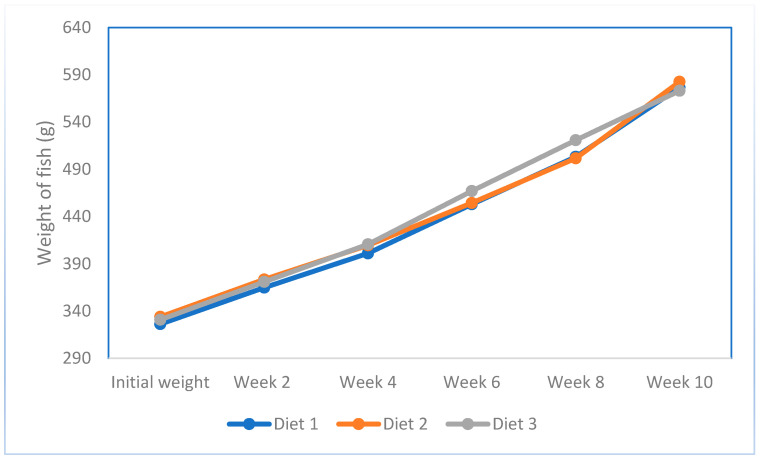
Weight of sobaity seabream versus period (Experiment 1).

**Figure 2:**
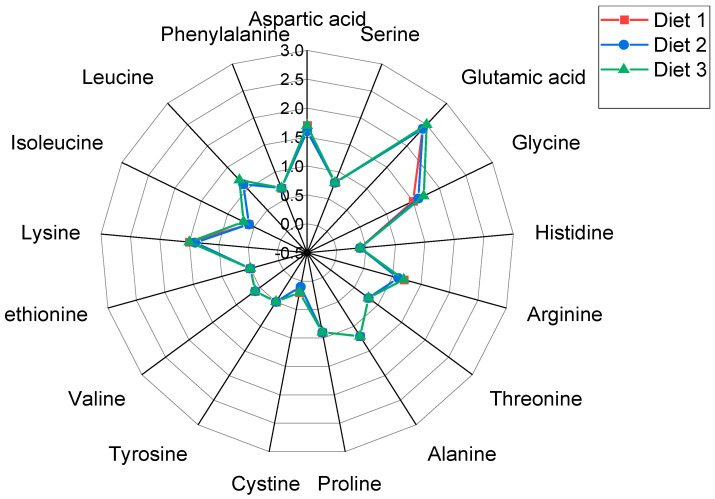
Radar plot of the amino acid profile (% dry matter) of commercial diets of sobaity seabream for Experiment 1.

The growth performance and survival of fish fed the different diets is presented in Table 5. Survival was not significantly different among the three diets tested. No significant differences were observed in the final body weight (Figure 1), with final weight gain, daily weight gain, and specific growth rate (SGR) similar across all three commercial diets. The final body weight of the fish after 80 days of culture was 576.6 g for diet 1, 582.6 g for diet 2, and 573.1 g for diet 3. The observed SGRs were 0.68, 0.69, and 0.68% day^−1^ for diets 1, 2, and 3, respectively. The FCR was significantly lower (*p* < 0.05) in fish fed diet 2 (1.42) compared to the groups fed diet 1 (1.61) and diet 3 (1.63). The PER was significantly higher in diet 2 (1.54) compared to diet 1 (1.41) and diet 3 (1.37). No significant differences were observed in the total feed intake among the three diets. Feed intake (FI) by the fish fed each of the three commercial diets was around in the range of 348.3–399.6 g fish^−1^ with the lowest FI by fish fed diet 2. After 80 days of culture, the fish fed diet 1 attained a final stocking density of 19.4 kg m^−3^, while those fed diet 2 reached 19.6 kg m^−3^, and those on diet 3 reached 19.1 kg m^−3^. The carcass composition was not significantly different between groups (Table 6). The amino acid profile of the commercial diets did not show any significant differences among them (Figure 2).

Overall, our data from these preliminary studies show that the best growth and feed utilization in sobaity seabream was obtained at a water temperature of 28 °C with a diet containing 46% crude protein and 20 MJ/kg, a protein-to-energy ratio of 23 mg/kJ.

### 3.2. Experiment 2

The DADC values of dry matter, protein, lipid, starch, and energy of the reference (D1) and test diets (D2–D11) for sobaity are given in Table 7. The DADC for dry matter of the different diets varied from 44.2 to 64.2%, though averaged around 51 ± 5.2%. The apparent digestibility coefficients of crude protein and energy ranged between 82.3 and 91.9% and 66.8 and 81.2%, respectively, among the diets. The DADC values of lipid and starch for experimental diets were recorded in the range of 86.1 to 93.1% and 90.0 to 99.6%, respectively.

The IADC of dry matter, crude protein, lipid, starch, and energy of test ingredients is provided in Table 8. The IADC values for dry matter (93.7%) and crude protein (96.6%) of wheat gluten were significantly higher than those of the other protein ingredients tested. The lowest IADC value for dry matter (26.9%) and crude protein (58.7%) was noted in the wheat meal. However, the IADC values of dry matter for soybean meal (46.9%) and tuna meal (46.6%) were also similarly low. High-quality fish meal (LT70 Norvik) and SPC had similar (64%) IADC values for dry matter. The IADCs of dry matter for fish meal UMP (48.8%), corn gluten meal (52.7%), and soybean meal (50%, Arasco, Riyadh, Saudi Arabia), had similar values. The IADC of crude protein for soybean meal was 93.9%. The protein digestibility of tuna meal (81.5%), fish meal UMP (83.6%), and the high-quality fish meal (LT70 Norvik, 85.8%) were found to be similar for this fish species. However, corn gluten meal (86%), SPC (77.2%), and soybean meal (Arasco, 93%) showed substantial variation in their IADCs of crude protein. The IADC of crude protein (75.5%) was noted in fish meal (Arasco). The IADCs for crude lipids of the tested ingredients ranged from 58.6% to 92.9%, and the lowest and highest values were observed in the high-quality fishmeal 3 (LT70 Norvik) and soy protein concentrate (SPC, Soycomil), respectively. No significant differences were observed among the IADC values of lipids of ARASCO fish meal, tuna meal, and SPC. The IADCs of energy of tuna meal and SPC, at 85%, was significantly higher than other protein ingredients (*p* <  0.05), followed by high-quality fish meal (LT70 Norvik), wheat gluten, UMP fish meal, corn gluten meal, ARASCO fish meal, soybean meal, and wheat meal.

## 4. Discussion

Sobaity seabream, a native species in the Arabian Gulf, is considered one of the potential species of interest for aquaculture diversification in the region. As a key development requirement, feeds need to be optimized for both the species and environment as a prerequisite for industry development. In this study, therefore, the initial application of three commercial diets to assess general diet specifications was evaluated before assessing nutrient availability from a range of available ingredients to be later used in locally produced diet formulations.

The results from the first trial showed that the different commercial diets used did not significantly affect the growth or survival (98 to 99%) of the fish but did significantly affect the feed conversion ratio and protein efficiency ratio. Fish fed the commercial diet with the highest protein and energy density (diet 2) showed better FCR (1.42) and PER (1.54) compared with other diets, which may be due to the higher energy content of diet 2 leading to a lower feed conversion ratio. In this trial, fish showed a maximum daily weight gain index of 1.74% and an SGR of 0.69% day^−1^. Another study [4] reported SGR values (0.62–0.76% day^−1^) similar to this study. Sobaity seabream have been reported with an SGR of around 1.2–1.4% day^−1^ in another study [17]. The variable SGR values may be because of variations in the initial size, temperature, and duration of the trials. The growth indicators obtained in this study suggest that sobaity seabream can grow well in the Red Sea conditions using these commercial diets.

The trial also showed that the different commercial diets used were well accepted by the fish. Feed intake was higher, though not significantly, for some diets, and it was hypothesized that this could be due to the differences in energy content in the diets. It has been observed with other fish species that the energy density of the feed directly influences the amount of feed consumed, such that a higher-energy-density diet can result in a reduction in the amount of food eaten by the fish [18]. Lipids are the most energetically dense substrate of all the dietary nutrients, which typically provide almost double the energy of protein and more than double that from carbohydrates. To date, the optimum dietary lipid requirement for sobaity seabream juveniles has yet to be investigated [19], and we suggest this as an important piece of work to follow up on.

Given the importance of dietary protein to fish maintenance, growth, and health, optimizing the dietary protein level and quality is another important step in diet development for this species. Optimal protein content in the diet has been shown to vary with diet energy density as well as the size of the fish [20]. In the case of sobaity seabream, few studies have investigated the optimum protein requirement. In a previous study with small sobaity seabream (28 g), a higher growth rate was observed in fish fed a diet containing 60% protein when compared to diets containing 45% and 50% protein levels [21], showing that the species requires high protein diets at this size. The study also recommended 50% protein with 22 MJ kg^−1^ energy at a P:E ratio of 23 g/MJ to cover its protein and energy requirements. In this study, the various commercial diets tested had only a limited range (44 to 46%) in their protein, while the energy content was also similar at around 19.7 to 20.1 MJ kg^−1^. It is suggested that these diets could be improved by optimizing the nutritional characteristics to better meet the dietary nutritional requirements of the species. No information was available on the feed ingredients used in the commercial diets, but alternative protein sources with better digestibility and palatability characteristics should be explored to produce an economically feasible diet while not compromising the growth of the fish.

In the second experiment, a variety of feed ingredients were evaluated for their apparent digestibility coefficients. Since fish meal is a limited protein source, the development of alternative, low-cost protein ingredients for use by the aquafeed industry remains a priority. Because protein is generally the most expensive component in feed formulations [9], obtaining information on the apparent digestibility coefficients (ADCs) of ingredients is required to formulate lower-cost feeds meeting the nutrient requirements for each cultured species. The use of low-cost ingredients may help lower feed costs, and in addition, the use of renewable protein ingredients should help achieve more sustainable aquaculture feeds [22].

In this study, the IADC of crude protein for soybean meal ranged from 92.8 to 93.9%. The digestibility of the various fish meals ranged from 75.5% to 85.8%, with the ARASCO fish meal (75.5%), tuna meal (81.5%), fish meal UMP (83.6%), and the high-quality fish meal (LT70 Norvik, 85.8%), all being among the mid-range of the ingredients examined. Corn gluten meal (86%) and SPC (77.2%) showed substantial differences from each other in their protein digestibility. The IADCs of lipid in test ingredients varied from 58.6% to 92.9% for sobaity seabream in this study but are notably notoriously difficult to determine accurately due to the low levels in the test ingredients and the low levels in the feces exacerbating error [11]. The IADCs of similar feed ingredients to those used in this study, but for other fish species, have been given in Table 9. For the juvenile South American characin dourado, *Salminus brasiliensis* (19.5 g), for instance, Ref. [23] recorded ADCs of 94.3% and 91% for protein and energy, respectively; juvenile rockfish, *Sebastes schlegeli* (30 g), had ADCs of 88% and 90% for protein and energy of white fish meal, respectively, and 92% and 93% for protein and energy of anchovy meal, respectively [24]; juvenile cobia, *Rachycentrum canadum* (10 g), had ADCs of 96.3% and 95.5% for protein and energy of Peruvian fish meal, respectively [25]; juvenile striped bass, *Morone saxatilis* × *Morone chrisops* (50 g), averaged ADCs of 88.2% and 95.6% for protein and energy of menhaden meal, respectively [26]; juvenile grouper, *Epinephelus coioides* (12 g), had, respectively, ADCs of 89.9% and 93.3% for protein and energy of white fish meal, and 87.4% and 89.5% for protein and energy of brown fish meal [27]; and, finally, juvenile yellowfin seabream, *Sparus latus* (41 g), had ADCs for protein and energy of 86.4% and 93.6%, respectively, for white FM [28]. The variation in ADCs was suggested to be a key indicator of the quality of the ingredients.

The IADCs of protein of SBM for sobaity in this study were 93%, which is comparable to those recorded for other carnivorous fish species, such as 95% with Japanese sea bass [35] and 90.9% with gilthead seabream [32]. Many factors are known to influence the digestibility of a given dietary ingredient. Among those criteria are the type of ingredient and the degree of processing utilized to produce it, as well as the source and quality of the original raw material used [32,39,40].

## 5. Conclusions

In conclusion, the IADCs of dry matter, protein, lipid, and energy in the various test ingredients fed to sobaity seabream differed substantially among the different ingredients. These differences in the IADCs of nutrients and energy may be explained by the differences in chemical composition, origin, and processing of these various ingredients. The characterization and determination of the IADCs of these ingredients allow for their judicious application to feeds for this species now that we have these data. Sobaity seabream also exhibited good growth performance and feed efficiency in the Red Sea conditions when fed a series of non-optimized diets, showing that it is a suitable species for aquaculture diversification in the region and may have scope for further optimization of its growth and feed efficiency. Both survival and growth of sobaity seabream cultured under Red Sea conditions were high, and further improvement and targeted studies on optimizing the nutritional characteristics of the feed are essential to continue improving the performance of the species.

## Figures and Tables

**Table 1:** Proximate composition and gross energy of the commercial diets (% dry matter unless indicated) used in Experiment 1.

Proximate	Diet 1	Diet 2	Diet 3
Dry Matter	91.4	92.6	93.4
Protein (*N ×* 6.25)	44.2	46.4	45.5
Lipid	11.7	12.5	11.9
Ash	8.4	9.7	8.9
Carbohydrate *	27.1	24.1	27.1
Gross energy (MJ kg^−1^)	19.74	20.06	20.13
P/E ratio (g MJ^−1^)	22.36	23.11	22.62
NPE (non-protein energy, MJ kg^−1^)	9.19	8.98	9.27

* Carbohydrate (CHO) content determined based on dry matter minus protein, ash, and lipids (CHO% = dry matter − (crude protein + crude ash + crude lipid)).

**Table 2:** Composition of macro and micro minerals in the commercial diets (% or mg 100 g^−1^ DM) used in Experiment 1.

	Diet 1	Diet 2	Diet 3
%			
Calcium	1.728	2.303	2.195
Magnesium	0.233	0.196	0.225
Phosphorus	1.203	1.521	1.422
Potassium	1.264	0.953	0.961
Sodium	0.142	0.263	0.397
Sulfur	0.415	0.535	0.603
mg 100 g^−1^			
Zinc	17.48	14.39	25.98
Copper	4.16	1.64	5.78
Iron	21.34	27.77	26.50
Manganese	3.32	10.29	4.23
Aluminum	3.80	7.69	7.05
Selenium	0.10	0.20	0.20
Cadmium	0.02	0.03	0.04
Chromium	0.04	0.09	0.07
Cobalt	<0.01	0.10	0.15
Lead	0.03	0.02	0.02
Molybdenum	0.29	0.18	0.14
Nickel	0.17	0.09	0.14
Tin	<0.01	<0.01	<0.01

**Table 3:** Test ingredients and their chemical compositions used in Experiment 2 (% dry matter).

	FM-UMP	FM-ARAS	FM LT70	Tuna Meal	Corn Gluten Meal	Soybean Meal (Sparos)	Soybean Meal (Arasco)	Wheat Flour	Wheat Gluten Meal	Soy Protein Concentrate (Soycomil)
Dry matter (%)	93.6	94	92.5	92.5	93.9	90.8	91.1	90.1	93.9	92.1
Protein (% DM)	56.9	59.3	60	50.4	58.4	43.5	41.9	11.9	73.2	62.4
Lipid (% DM)	9.19	9.12	7.65	10.2	9.22	2.68	3.28	2.59	7.53	0.4
Energy (kJ/g DM)	19.74	18.00	19.77	18.86	22.90	19.65	19.77	18.33	23.44	18.00

**Table 4:** Diet formulations and chemical compositions of basal and test diets for Experiment 2 (all values are % as used unless otherwise indicated).

Ingredients	Reference Diet D1	Test Diets D2 to D11
Fishmeal-Superprime	20	14
Sardine Oil	7.5	5.3
Wheat Meal	16.3	11.4
Corn Gluten	15	10.5
Wheat Gluten	15	10.5
Soy Protein Concentrate	25	17.5
Proteinaceous Test Ingredients	0	30
Lipid Test Ingredients	0	0
Vit and Min Premix	1	0.7
Choline	0.1	0.07
Yttrium Oxide	0.1	0.07
Diet Composition (indicative ranges)		
Dry Matter	93	92–94
Crude Protein	45.6	34.5–53.3
Lipids	11.9	8.7–12.4
Ash	6.1	1.1–10.9
Carbohydrates *	29.4	21.8–46.1
Gross Energy (kJ g^−1^)	20.5	19.8–21.6

* Carbohydrate content determined based on dry matter minus protein, ash, and lipids (CHO% = dry matter − (crude protein + crude ash + crude lipid)).

**Table 5:** Survival and growth parameters of sobaity seabream fed different locally available commercial diets (Experiment 1).

	Diet 1	Diet 2	Diet 3	ANOVA *p*-Value
Survival (%)	99.0 ± 1.2	99.0 ± 1.2	98.0 ± 1.2	0.729
Initial body weight (g)	326.9 ± 1.0	333.8 ± 3.1	330.8 ± 1.4	0.063
Final body weight (g)	576.6 ± 3.2	582.6 ± 9.4	573.1 ± 2.3	0.833
Initial total length (cm)	26.8 ± 0.3	26.9 ± 0.1	26.9 ± 0.2	0.165
Final total length (cm)	31.8 ± 0.2	32.0 ± 0.3	31.8 ± 0.2	0.780
Final stocking density (kg m^−3^)	19.4 ± 0.3	19.6 ± 0.5	19.09 ± 0.3	0.594
Total weight gain (g)	249.8 ± 2.6	248.8 ± 1.4	242.3 ± 3.7	0.855
Daily growth index (% day^−1^)	1.74 ± 0.2	1.71 ± 0.2	1.68 ± 0.1	0.773
Specific growth rate (% day^−1^)	0.68 ± 0.01	0.69 ± 0.02	0.68 ± 0.01	0.760
Thermal growth coefficient (% degree-day^−1^)	0.63 ± 0.02	0.62 ± 0.02	0.4 ± 0.02	0.760
Feed intake (g fish^−1^)	399.6 ± 4.1	348.3 ± 2.5	387.6 ± 6.8	0.085
Feed conversion ratio	1.61 ± 0.0 ^a^	1.42 ± 0.0 ^b^	1.63 ± 0.0 ^a^	0.003
Protein efficiency ratio	1.41 ± 0.0 ^b^	1.54 ± 0.1 ^a^	1.37 ± 0.0 ^b^	0.017

Values (means ± SEM, *N* = 3) within a row with a common superscript letter are not significantly different from the other dietary groups (*p* > 0.05).

**Table 6:** Carcass composition (% wet basis) of sobaity seabream fed different locally available commercial diets (Experiment 1).

	Diet 1	Diet 2	Diet 3	ANOVA *p*-Value
Moisture	69.5 ± 6.3	70.4 ± 6.3	68.2 ± 6.3	0.137
Crude protein	18.7 ± 0.5	19.1 ± 0.5	18.4 ± 0.5	0.251
Crude fat	6.9 ± 0.2	6.2 ± 0.2	7.1 ± 0.2	0.165
Ash	3.9 ± 0.08	4.2 ± 0.08	4.0 ± 0.08	0.321

Values are the means of triplicate samples (means ± SEM, *N* = 3).

**Table 7:** Diet apparent digestibility coefficient (DADC) values of basal (D1) and test diets (D2–D11) of sobaity seabream (Experiment 2).

Diets	Test Ingredients	Dry Matter (ADC%)	Protein (ADC%)	Lipid (ADC%)	Starch (ADC%)	Energy (ADC%)
D1	Reference Diet	51.1 ± 2.6	86.8 ± 3.1	93.1 ± 2.8	98.0 ± 5.1	74.2 ± 2.3
D2	Fish Meal (UMP)	50.2 ± 1.9	83.3 ± 1.9	90.3 ± 4.1	99.2 ± 3.5	75.4 ± 2.8
D3	Fish Meal (ARASCO)	45.4 ± 0.6	82.3 ± 5.3	90.6 ± 1.8	99.4 ± 2.4	69.5 ± 2.1
D4	Fishmeal 3 (LT70 Norvik)	54.1 ± 5.1	85.4 ± 3.6	92.3 ± 3.4	99.6 ± 4.1	76.4 ± 4.5
D5	Tuna Meal	49.2 ± 3.2	82.2 ± 1.6	92.3 ± 3.1	99.3 ± 4.5	74.0 ± 4.2
D6	Corn Gluten Meal (ARASCO)	51.3 ± 2.9	84.9 ± 2.9	90.9 ± 3.6	98.1 ± 4.3	73.8 ± 3.7
D7	Soybean Meal	49.6 ± 4.6	87.8 ± 4.8	89.7 ± 4.5	99.0 ± 3.7	71.3 ± 2.9
D8	Soybean Meal (ARASCO)	51.0 ± 3.1	88.1 ± 3.6	87.0 ± 3.8	97.7 ± 3.4	71.8 ± 1.6
D9	Wheat Meal	44.2 ± 2.5	87.0 ± 2.9	89.4 ± 2.9	90.0 ± 3.9	66.8 ± 1.1
D10	Wheat Gluten	64.2 ± 2.4	91.9 ± 1.5	86.1 ± 2.5	99.0 ± 3.1	81.2 ± 1.5
D11	Soy Protein Concentrate (SPC, Soycomil)	54.7 ± 1.1	87.1 ± 4.2	90.3 ± 3.6	98.1 ± 2.8	73.6 ± 3.5
	Overall Mean	51.38 ± 5.29	86.09 ± 2.86	90.19 ± 2.26	97.98 ± 2.72	73.48 ± 3.75
	ANOVA *p*-Value	*p* < 0.05	*p* < 0.05	*p* < 0.05	*p* < 0.05	*p* < 0.05

Values are the means of triplicate samples (±SEM, *N* = 3), ADC; apparent digestibility coefficient.

**Table 8:** The ingredient digestibility (IADC) values for the tested ingredients (D2–D11) fed to sobaity seabream (Experiment 2).

Test Diets	Test Ingredients	Dry Matter (IADC%)	Protein (IADC%)	Lipid (IADC%)	Energy (IADC%)
D2	Fish Meal (UMP)	48.8 ± 0.8	83.6 ± 1.9	79.1 ± 3.1	78.1 ± 1.4
D3	Fish Meal (ARASCO)	33.5 ± 1.1	75.5 ± 1.2	91.1 ± 2.9	75.2 ± 1.1
D4	HQ Fishmeal (LT70 Norvik)	63.6 ± 1.8	85.8 ± 2.4	58.6 ± 0.9	84.5 ± 2.6
D5	Tuna Meal	46.6 ± 0.7	81.5 ± 1.8	90.7 ± 2.1	85.2 ± 2.1
D6	Corn Gluten Meal (ARASCO)	52.7 ± 0.5	86.0 ± 2.8	83.7 ± 2.3	77.7 ± 3.4
D7	Soybean Meal	46.9 ± 0.2	93.9 ± 3.1	78.4 ± 1.7	67.9 ± 1.8
D8	Soybean Meal (ARASCO)	50.0 ± 1.1	92.8 ± 2.9	72.0 ± 1.4	70.7 ± 1.1
D9	Wheat Meal	26.9 ± 1.7	58.7 ± 2.4	62.6 ± 1.1	50.6 ± 0.9
D10	Wheat Gluten	93.7 ± 1.2	96.6 ± 2.6	57.4 ± 2.6	82.2 ± 2.8
D11	Soy Protein Concentrate (SPC, Soycomil)	64.0 ± 0.8	77.2 ± 4.1	92.9 ± 2.3	85.0 ± 1.7
	ANOVA *p*-Value	*p* < 0.05	*p* < 0.05	*p* < 0.05	*p* < 0.05

Values are the means of triplicate samples (±SEM, *N* = 3) IADC; Ingredient apparent digestibility coefficient.

**Table 9:** The ingredient digestibility (%IADC) values for similar ingredients with other fish species.

Feed Ingredients	Fish Meal	Tuna Meal	Corn Gluten Meal	Soybean Meal	Wheat Meal	Wheat Gluten	Soy Protein Concentrate (SPC)
**%IADC**	Gilthead seabream (*Sparus aurata*)						
**Dry matter**	70.3 ^1^		-	-	-	-	-
**Protein**	84.6 ^2^		90 ^3^	90.9 ^4^	80 ^5^	96 ^3^	92 ^3^
**Lipid**	79.7 ^2^		82.9 ^4^	62.9 ^4^	99 ^5^	-	-
**Energy**	84 ^3^		72 ^3^	44.7 ^4^	91.8 ^6^	91 ^3^	75 ^3^
	Japanese seabass (*Lateolabrax japonicas*)						
**Dry matter**	83.14 ^7^			80.01 ^7^			
**Protein**	91.18 ^7^			95.04 ^7^			
**Energy**	91.14 ^7^			83.22 ^7^			
	Asian seabass (*Lates calcarifer*)						
**Dry matter**	49–75.68 ^8,10^	63.2 ^8^	62.1 ^9^	31–64.1 ^8,10^	51.7 ^8^		53–68.4 ^8,10^
**Protein**	71–85.2 ^8,10^	77.8 ^8^	88.5 ^9^	68–81.0 ^8,10^	74.1 ^8^		49–95.9 ^8,10^
**Lipid**	45–94.9 ^8,10^	94.9 ^8^	70.0 ^9^	57–87.5 ^8,10^	94.1 ^8^		85–89.2 ^8,10^
**Energy**	56–84.7 ^8,10^	79.3 ^8^	73.2 ^8^	35–74.3 ^8,10^	63.5 ^8^		49–77.5 ^8,10^

^1^ [29], ^2^ [30], ^3^ [31], ^4^ [32], ^5^ [33], ^6^ [34], ^7^ [35], ^8^ [36], ^9^ [37], ^10^ [38].

## Data Availability

The data used in the outcomes of this research are included in this manuscript.

## Abbreviations

ANOVA, analysis of variance; AWG, average body weight gain; FCR, feed conversion ratio; SGR, specific growth rate; PER, protein efficiency ratio.

## References

[1] Kitto M. (2004). Sobaity–the Arab’s choice. Fish Farming.

[2] Al-Asheeri S., Freije A., Perna S. (2020). Farmed Versus Wild Fish Consumption in Relation to Fatty Acid Composition in the Kingdom of Bahrain. Egypt. J. Aquat. Biol. Fish..

[3] Azhdari N., Kochnine P., Zakeri M., Yavari V. (2019). Effects of dietary protein and lipid levels on growth performance, feed utilization and carcass biochemical composition in *Sobaity juvenile*, *Sparidentex hasta*. J. Fish..

[4] Hekmatpour F., Kochanian P., Marammazi J.G., Zakeri M., Mousavi S.-M. (2019). Changes in serum biochemical parameters and digestive enzyme activity of juvenile sobaity sea bream (*Sparidentex hasta*) in response to partial replacement of dietary fish meal with poultry by-product meal. Fish Physiol. Biochem..

[5] Hossain M., Al-Abdul-Elah K., Yaseen S. (2019). Seasonal variations in proximate and fatty acid composition of sobaity sea bream (*Sparidentex hasta*) in Kuwait waters. J. Mar. Biol. Assoc. UK.

[6] Hossain M., Al-Adul-Elah K., Azad I., Alzalzalah A., Alnuiami S. (2022). High DHA Algae Meal as Cost-effective Alternative to High DHA Fish Oil in Finisher Feed for Sobaity Sea Bream (*Sparidentex hasta*). Anim. Feed Sci. Technol..

[7] Hossain M.A., Al-Abdul-Elah K., El-Dakour S. (2019). Improvement of nutritional quality of cultured sobaity sea bream, *Sparidentex hasta* (Valenciennes) muscle by preharvest feeding of finisher feeds. J. Appl. Ichthyol..

[8] Yaghoubi M., Torfi Mozanzadeh M., Ghafleh Marammazi J., Omid Safari O., Gisbert E. (2019). Effects of soy protein base diet supplemented with lysine and methionine on digestive enzymes activity and hematological parameters in silvery-black porgy (*Sparidentex hasta*) juveniles. Iran. J. Fish. Sci..

[9] Tacon A.G., Hasan M.R., Metian M. (2011). Demand and Supply of Feed Ingredients for Farmed Fish and Crustaceans: Trends and Prospects.

[10] Yang Q., Zhou X., Zhou Q., Tan B., Chi S., Dong X. (2009). Apparent digestibility of selected feed ingredients for white shrimp *Litopenaeus vannamei*, Boone. Aquac. Res..

[11] Glencross B.D. (2020). A feed is still only as good as its ingredients: An update on the nutritional research strategies for the optimal evaluation of ingredients for aquaculture feeds. Aquac. Nutr..

[12] APHA (1992). Standard Methods for the Examination of Dairy Products.

[13] AOAC (2005). Association of Official Analytical Chemists.

[14] Glencross B., Hawkins W. (2004). A comparison of the digestibility of lupin (*Lupinus* sp.) kernel meals as dietary protein resources when fed to either rainbow trout, *Oncorhynchus mykiss* or red seabream *Pagrus Auratus*. Aquac. Nutr..

[15] Glencross B.D., Booth M., Allan G.L. (2007). A feed is only as good as its ingredients—A review of ingredient evaluation strategies for aquaculture feeds. Aquac. Nutr..

[16] Elsdon T.S., Gillanders B.M. (2002). Interactive effects of temperature and salinity on otolith chemistry: Challenges for determining environmental histories of fish. Can. J. Fish. Aquat. Sci..

[17] Hossain M., Al-Abdul-Elah K., El-Dakour S. (2014). Evaluation of different commercial feeds for culture of juvenile sobaity (*Sparidentex hasta* Valenciennes) in Kuwait. APCBEE Procedia.

[18] Glencross B., Blyth D., Irvin S., Bourne N., Wade N. (2014). An analysis of the effects of different dietary macronutrient energy sources on the growth and energy partitioning by juvenile barramundi, *Lates calcarifer*, reveal a preference for protein-derived energy. Aquac. Nutr..

[19] Torfi Mozanzadeh M., Marammazi J.G., Yaghoubi M., Agh N., Pagheh E., Gisbert E. (2017). Macronutrient requirements of silvery-black porgy (*Sparidentex hasta*): A comparison with other farmed sparid species. Fishes.

[20] Glencross B. (2006). The nutritional management of barramundi, *Lates calcarifer*—A review. Aquac. Nutr..

[21] Ghofleh Marammazi J., Najafabadi M., Pagheh E., Hafezieh M. (2017). Effects of Varying Levels of Dietary Protein and Energy on the Growth, Food Performance and Body composition of Sobeity (*Sparidentex hasta*) Juvenile. J. Mar. Sci. Technol..

[22] Booth M., Allan G., Smullen R. (2013). Digestibility of common feed ingredients by juvenile mulloway *Argyrosomus japonicus*. Aquaculture.

[23] Borghesi R., Dairiki J., Cyrino J.E.P. (2009). Apparent digestibility coefficients of selected feed ingredients for dourado *Salminus brasiliensis*. Aquac. Nutr..

[24] Lee S.-M. (2002). Apparent digestibility coefficients of various feed ingredients for juvenile and grower rockfish (*Sebastes schlegeli*). Aquaculture.

[25] Zhou Q.-C., Tan B.-P., Mai K.-S., Liu Y.-J. (2004). Apparent digestibility of selected feed ingredients for juvenile cobia *Rachycentron canadum*. Aquaculture.

[26] Sullivan J.A., Reigh R.C. (1995). Apparent digestibility of selected feedstuffs in diets for hybrid striped bass (*Morone saxatilis* ♀ x *Morone chrysops* ♂). Aquaculture.

[27] Lin H., Liu Y., Tian L., Wang J., Zheng W., Huang J., Chen P. (2004). Apparent digestibility coefficients of various feed ingredients for grouper *Epinephelus coioides*. J. World. Aquac. Soc..

[28] Wu X.Y., Liu Y.J., Tian L.X., Mai K.S., Yang H.J. (2006). Apparent digestibility coefficients of selected feed ingredients for yellowfin seabream, *Sparus latus*. J. World. Aquac. Soc..

[29] Mastoraki M., Panteli N., Kotzamanis Y.P., Gasco L., Antonopoulou E., Chatzifotis S. (2022). Nutrient digestibility of diets containing five different insect meals in gilthead sea bream (*Sparus aurata*) and European sea bass (*Dicentrarchus labrax*). Anim. Feed Sci. Technol..

[30] Mabrouk H., Nour A. (2011). Assessment of apparent digestibility coefficients (ADCs%) of some animal protein sources by gilthead sea bream (*Sparus aurata*). Egypt. J. Aquat. Res..

[31] Kissil G.W., Lupatsch I. (2004). Successful replacement of fishmeal by plant proteins in diets for the gilthead seabream, *Sparus aurata* L.. Isr. J. Aquac.-Bamidgeh.

[32] Nengas I., Alexis M., Davies S., Petichakis G. (1995). Investigation to determine digestibility coefficients of various raw materials in diets for gilthead sea bream, *Sparus auratus* L.. Aquac. Res..

[33] Couto A., Peres H., Oliva-Teles A., Enes P. (2016). Screening of nutrient digestibility, glycaemic response and gut morphology alterations in gilthead seabream (*Sparus aurata*) fed whole cereal meals. Aquaculture.

[34] Venou B., Alexis M., Fountoulaki E., Haralabous J. (2006). Effects of extrusion and inclusion level of soybean meal on diet digestibility, performance and nutrient utilization of gilthead sea bream (*Sparus aurata*). Aquaculture.

[35] Wang J., Yun B., Xue M., Wu X., Zheng Y., Li P. (2012). Apparent digestibility coefficients of several protein sources, and replacement of fishmeal by porcine meal in diets of Japanese seabass, *Lateolabrax japonicus*, are affected by dietary protein levels. Aquac. Res..

[36] Booth M.A., Pirozzi I. (2021). The digestibility of raw materials by barramundi Lates calcarifer: Emphasis on the effect of inclusion rate on the digestibility of soybean meal and soy protein concentrate. Anim. Feed Sci. Technol..

[37] Nandakumar S., Ambasankar K., Ali S.S.R., Syamadayal J., Vasagam K. (2017). Replacement of fish meal with corn gluten meal in feeds for Asian seabass (*Lates calcarifer*). Aquac. Int..

[38] Glencross B., Blyth D., Cheers S., Bourne N., Wade N., Irvin S. (2017). A compendium of raw material digestibilities for barramundi, Lates calcarifer. Aquac. Nutr..

[39] Rahman M.M., Kim K.-W., Lee S.-M. (2022). Apparent digestibility coefficients of animal feed ingredients for olive flounder (*Paralichthys olivaceus*). Fish. Aquat. Sci..

[40] Madrid J., Pohlenz C., Viana M.T., Lazo J.P. (2023). Apparent digestibility coefficients of selected protein ingredients for juvenile *Totoaba macdonaldi*. J. World Aquac. Soc..

